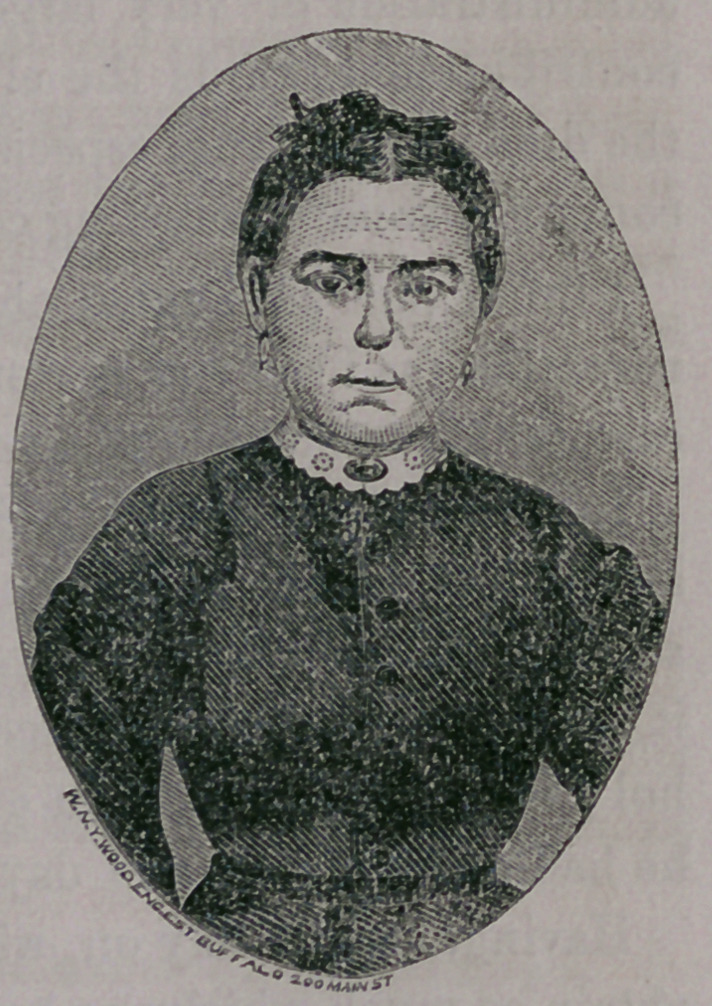# Rhinoplasty—Talicotian Operation

**Published:** 1871-09

**Authors:** J. F. Miner


BUFFALO

Medical and Surgical Journal.

VOL. XI. SEPTEMBER, 1871. No. 2.

### Original Communications.

ART. I. *Rhinoplasty—Talicotian Operation. Result Illustrated.* Chinical Lecture by PROF. J. F. MINER. Reported by W. W. MINER, member of the Class of 1870-'71.

GENTLEMEN:—

I have a Patient upon whom I propose to operate this morning for the restoration of the nose. Operations for restoring the form of the nasal organ are called *Rinoplastic.* Two procedures have been employed, viz: 1st, the *Talicotian* operation, or Italian, from being the country of Talicotius, which consists in taking the integument and areolar tissue required for the repair of the lost organ from the arm, and; 2nd, the *Indian,* which consists in taking the required tissue from the forehead. The operation as first practised by Talicotius, chiefly in consequence of the tedious and painful confinement of the head and arm, and the uncertainty of its results is now seldom practiced; the integument required is, at present, generally borrowed from the adjacent parts, and twisted or slid into position as found necessary, no one plan of procedure being applicable in all cases.

The young lady before you has lost the entire lower half of this organ, leaving the nasal cavity exposed and unsightly. Into the nature of this accident we do not propose this morning to inquire, for reasons sufficiently obvious.

After three years treatment for the disease which caused this unsightly deformity, it is believed that the parts and system generally have so far recovered as to make operation for restoration feasable.

The reasons for making an operation now nearly abandoned on account of the great difficulties of the undertaking require brief mention.

When sufficient integument is taken from the forehead to answer the purpose of repair, we have made so great a deformity on the forehead of the young lady that little would be gained towards restoring the natural appearances of the face. To take the tissue from the forehead is easier of execution, more certain of success, less painful from unnatural constraint and on many accounts to be chosen, the fatal objection to my mind, being the *deformity* it causes. It might be nearly as well to obtain the gutta purcha nose now manufactured, rather than attempt to make a nose at the sacrifice of the forehead. However, if I was obliged to choose, I would try the Indian operation rather than wear the artificial, made of rubber or papier mache. The paring of the parts, the right proportion, size, shape, and adaptation of the flap, require some of the nicest processes of surgery, and when these are employed, you are by no means certain of very satisfactory results, causes not under the control of the Surgeon may thwart your well directed efforts; partial success only is the rule; improvement may be considered success, increased deformity, failure.

We cannot now review the points of interest in detail, but must show you the operation, and describe the processes as we proceed. We first pare the soft parts at the line where we propose to attach our material of repair, taking care to remove the hardened edge so as to leave a healthy surface, full of vessels in active circulation, then a flap is dissected from the back of the arm 3 1-2 inches wide by 4 inches long, going down in this case to the superficial fascia of the arm. This flap is made in shape to acurately conform to the required location, and in this, much careful calculation is necessary. After the bleeding has ceased the arm is brought up to the face and secured by adhesive straps. Talicotius probably did not have adhesive plaster, and instead, had an expensive apparatus not half so convenient or efficient. The flap is now carefully adjusted by its free extremity to the pared edges of the nose, and fastened by silver sutures, making calculation that the flap will shrink nearly one half in its size before the union becomes complete. It was formerly deemed necessary to raise the flap from the arm and let it contract, become vascular, &c., by an interval of ten days or more, when it was refreshened and adapted to the new situation. This might work very well, but we prefer to adapt it immediately, allowing for the necessary contraction, and let this process go on at the same time that adhesions between the nose and flap are forming. This dressing, holding arm to the head, and flap in place, we design to leave as you now observe it, for from nine to twelve days, according to appearances, when we must trust for life to newly formed vessels at the point of attachment.

Three months after the operation I again introduce the patient that you may observe and admire the perfection of the result. We have had many discouragements, and the case has required and received constant watching at the hands of the ever faithful and ever attentive Sister, in charge of this Ward, and as the result of this fidelity, we are able to show you a restored organ so perfect in all respects as not to attract attention as presenting anything unnatural. It has been examined by many Surgeons of great experience, both in our own and foreign countries, who pronounce the result, in all respects, unsurpassed. This experience leads me to believe that the Talicotian operation is very difficult and uncertain, and that, though successful in this instance in a remarkable degree, yet many partial, or entire failures, will accompany every such case of success. The risks of non-union, the liability to death of flap after separating it from the arm, the certainty that any change or pressure upon the delicate vessels which supply the part attached will interrupt the circulation, or destroy the vessels altogether, constitute sources of entire failure ; while miscalculation in the size, shape, and adjustment of the new material, is liable to cause failure in obtaining proper contour of the organ. The whole procedure is attended by difficulties and uncertainties ; but if attended by success in one case, the achievement will amply compensate for several failures.

## Figures and Tables

**Figure f1:**